# Basal Tone, Muscle Memory and Denervation: Pathophysiological Foundations of Abdominal Wall Hernia Development and Recovery

**DOI:** 10.3390/pathophysiology33030069

**Published:** 2026-09-14

**Authors:** Zelimkhan G. M. Berikkhanov, Zakhar A. Akulov, Maria A. Sukhanova, Alexey L. Shestakov, Aleksey G. Kotelnikov, Andrey M. Nikolaev, Vadim S. Razumovsky, Evgeniy A. Tarabrin, Milena Yu. Ivanova, Sergey Yu. Muraviev

**Affiliations:** Sechenov First Moscow State Medical University (Sechenov University), 119048 Moscow, Russiaakulov_z_a@student.sechenov.ru (Z.A.A.); kotelnikov_a_g@staff.sechenov.ru (A.G.K.); ivanova_m_yu_1@staff.sechenov.ru (M.Y.I.); muravev_s_yu@staff.sechenov.ru (S.Y.M.)

**Keywords:** abdominal wall hernia, neuromuscular function, basal tone, motor coordination, muscle memory, hernia, prehabilitation

## Abstract

Modern abdominal wall hernia surgery is centered on the biomechanics of the fascial-aponeurotic framework and on intra-abdominal pressure (IAP) control; neuromuscular regulation of the wall remains outside perioperative planning. This review reframes the abdominal wall as a coordinated neuromuscular system and outlines a framework in which ventral herniation is hypothesized to represent a compensatory response to altered regulation of elevated IAP, while discoordinated contraction of the muscular layers is proposed as a potential predisposing factor. A structured literature search was performed in PubMed/MEDLINE, Embase and the Cochrane Library from inception to March 2026 across four thematic blocks: basal tone and innervation, spinal pathology and coordination, skeletal muscle memory, and electromyographic and clinical models of denervation. Synthesis followed SANRA criteria. This is not a formal systematic review or meta-analysis. Basal tone of the abdominal muscles is individual and depends on spinal innervation, lifestyle, comorbidity, and hernia duration; obstructive pulmonary disease yields a hypertonic phenotype, and persistent meteorism a hypotonic one. In a patient predisposed to herniation, elevated IAP may be partially accommodated through redistribution of abdominal contents; within the proposed framework, herniation is hypothesized to function as a pressure-relief mechanism. Discoordination of the muscular layers may reduce the capacity of the abdominal wall to adapt to rapid or unfamiliar loading, potentially contributing to hernia formation in predisposed patients. Six meta-analyses of preoperative botulinum toxin type A confirm lateral muscle elongation; none assessed basal-tone recovery after toxin washout. Each hernia patient presents with an individual neuromuscular phenotype shaped by basal tone, intermuscular coordination and muscle memory. The proposed framework complements mechano-fascial concepts with a neurophysiological dimension and provides a hypothesis-generating rationale for investigating phenotype-tailored prehabilitation and postoperative rehabilitation. Prospective electromyographic mapping with functional maneuvers is the immediate research priority.

## 1. Introduction

Abdominal wall hernia surgery has developed intensively over three decades, standardizing mesh-based techniques and defect classifications [1,2,3]. The biomechanics of the fascial-aponeurotic framework have been studied in detail: intra-abdominal pressure (IAP), abdominal wall compliance, and volumetric relationships have been incorporated into routine preoperative planning [4,5]. However, most anatomically weak areas lack the mechanical strength to withstand IAP on their own and depend on active muscular protection. At the umbilicus, the linea alba is exposed to pressures of up to 230 mm Hg under maximal load [6]. However, the mechanical consequences of elevated IAP cannot be interpreted from pressure magnitude alone. According to Laplace-type biomechanical principles, tissue tension depends on both pressure and the local radius of curvature. Active medial approximation of the rectus muscles during contraction may therefore contribute not only to resisting IAP but also to maintaining a mechanically favorable configuration of the anterior abdominal wall. Within the neuromuscular framework proposed in this review, we hypothesize that alterations in basal tone and discoordinated contraction of individual muscle groups may modify local abdominal wall geometry and, consequently, the distribution of tissue tension during increases in IAP. This proposed relationship between neuromuscular coordination, abdominal wall geometry, and local mechanical loading remains to be directly demonstrated in patients with ventral hernias.

The neuromuscular component—basal tone, coordinated contraction of synergistic muscles, and adaptive remodeling of synapses—is not yet incorporated into clinical protocols. In current clinical practice, the neuromuscular status of the abdominal wall is not routinely evaluated before elective hernia repair, and rehabilitation is rarely planned according to the patient’s individual muscle tone. The mesh is placed, the fascia is closed, and the musculature is generally expected to recover without targeted neuromuscular retraining. Yet, adapted over time to the pathological state, these muscles cannot independently return to physiologically coordinated contraction without directed retraining.

This review examines the hypothesis that discoordinated muscular contraction, influenced by the individual state of basal tone, spinal innervation, and muscle memory, may contribute to ventral hernia formation; explores whether botulinum toxin and component separation, while addressing immediate surgical objectives, may leave aspects of neuromuscular coordination unresolved; and proposes phenotype-guided rehabilitation as a hypothesis-generating approach requiring prospective clinical validation.

## 2. Materials and Methods

### 2.1. Research Questions

#### 2.1.1. Pathogenesis

Is there electrophysiological or neurofunctional evidence of impaired basal tone, coordination, and adaptive neuromuscular remodeling in hernia patients compared with healthy individuals?

#### 2.1.2. Surgery and Rehabilitation

How do BTA-associated chemodenervation and surgical denervation during component separation affect the recovery of neuromuscular coordination, and what conceptual framework for phenotype-guided rehabilitation can be proposed for prospective evaluation?

### 2.2. Literature Search and Evidence Synthesis

This work was prepared as a conceptual narrative review with evidence mapping and a research agenda. It is not a formal systematic review or meta-analysis. The conceptual synthesis was guided by the SANRA (Scale for the Assessment of Narrative Review Articles) criteria. Literature was retrieved through a structured search strategy with predefined thematic blocks. The results were synthesized qualitatively, with the strength of evidence for each line of argument explicitly characterized (Table 1).

### 2.3. Research Design

A structured literature search was performed in PubMed/MEDLINE, Embase and the Cochrane Library from inception to March 2026, and results were restricted to the English language. The search combined free-text terms and MeSH headings organized into four thematic blocks. Block A (basal tone and denervation): (“botulinum toxin” AND “abdominal wall” AND (“hernia” OR “abdominal wall reconstruction”)), (“component separation” AND “denervation”), (“transversus abdominis release” AND “innervation”). Block B (spine and abdominal musculature): (“thoracolumbar nerve” AND “abdominal wall”), (“spondylotic radiculopathy” OR “degenerative thoracolumbar spine disease” AND “abdominal muscle”), (“motor coordination” AND “trunk” AND “intra-abdominal pressure”). Block C (muscle memory): (“myonuclear” AND “muscle memory”), (“satellite cell” AND “hypertrophy” AND “detraining”), (“neuromuscular junction” AND “remodeling”). Block D (electromyographic and clinical models): (“electromyography” AND “abdominal muscle” AND “hernia”), (“pseudohernia” AND “denervation”), (“abdominal wall relaxation” AND “neuropathy”).

### 2.4. Inclusion and Exclusion Criteria

Included publication types were systematic reviews and meta-analyses; randomized controlled trials; prospective and retrospective cohort studies; cadaveric anatomical studies; case reports of segmental denervation of the abdominal wall; and fundamental reviews of skeletal muscle neurophysiology. Studies dedicated exclusively to neurological disease without any connection to abdominal wall surgery or pathology, conference abstracts without a full-text version, and pediatric-only studies were excluded.

### 2.5. Quality Selection and Evaluation Process

Title and abstract screening were performed by one author, with full-text assessment and final inclusion decisions taken collegially by all authors. For clinical sections, systematic reviews and meta-analyses with the largest sample sizes and most recent publication dates received priority; for pathophysiological sections, fundamental and narrative reviews were used. The reference list contains only sources cited in the body of the text.

For the conceptual evidence map (Table 4), the evidence status of each proposition was classified using predefined operational criteria developed for the purposes of this review. “Supported by available evidence” was assigned when the proposition itself was directly addressed by clinical, experimental, or synthesized evidence relevant to the abdominal wall or the intervention under consideration. “Partially supported” indicated that direct evidence existed for some, but not all, components of the proposition, or that the available evidence did not establish the complete proposed relationship. “Indirectly supported” was assigned when the proposition was inferred from evidence addressing separate links of the proposed mechanistic chain or from findings extrapolated from adjacent populations, conditions, or physiological models, without direct demonstration of the complete relationship in ventral hernia patients. “Hypothesis only” indicated that no direct evidence for the complete proposition was identified and that the relationship represented a hypothesis derived from the conceptual synthesis. These categories were used for evidence mapping within this review and should not be interpreted as a formal evidence-grading system.

## 3. Results

### 3.1. Basal Tone as an Individual Characteristic of the Hernia Patient

Skeletal muscle at rest is not functionally silent. Spinal alpha-motor neurons maintain a continuous low-amplitude tonic discharge providing reflex readiness to contract via the alpha-gamma coactivation loop [7]. The transversus abdominis is activated in an anticipatory manner, 30–40 ms before the onset of voluntary limb movement, providing feed-forward trunk stabilization [8,9].

The basal tone of the abdominal wall muscles is dynamic and shaped by spinal innervation (Th7–L1 nerve roots), lifestyle, comorbidity, and the duration of hernia carriage. In a patient with obstructive pulmonary disease, the abdominal muscles adapt to a chronic expiratory pattern; in a patient with gastrointestinal disorders associated with persistent meteorism, a different profile develops. Every patient presents with a personal, gradually constructed neuromuscular phenotype.

The cause-and-effect relationship between IAP and hernia formation merits clarification: the notion that the hernia patient “lives in a state of chronically elevated IAP” is inconsistent with physiology. In a healthy subject, a rise in IAP is compensated by reflex relaxation of the muscular envelope. In a patient predisposed to hernia, this compensation may be impaired. Within the proposed framework, a persistent discoordinated contraction pattern could limit appropriate muscular relaxation and favor redistribution of abdominal contents toward an area of lower mechanical resistance, potentially contributing to defect formation. From this perspective, we hypothesize that herniation may, in some patients, represent a form of pressure-relieving redistribution rather than simply a direct consequence of chronically elevated IAP. This hypothesis may provide one possible explanation for the clinical observation that some patients with giant ventral hernias tolerate substantial extracavitary displacement of abdominal contents, whereas reduction in the hernia contents into the abdominal cavity may alter pressure-volume relationships. Within our conceptual model, a potential contributing mechanism is discoordinated contraction of the muscular layers: differences in activation amplitude and timing may produce transiently non-uniform mechanical loading of the abdominal wall. Progressive preoperative pneumoperitoneum represents a different biomechanical scenario, in which gradual expansion of the abdominal cavity may permit adaptation of the abdominal wall to increasing volume. How this differs from external reduction with an abdominal binder at the level of neuromuscular coordination remains insufficiently characterized. Progressive growth of a hernia may be accompanied by progressive alterations in neuromuscular coordination as hernia content volume increases; whether such discoordination contributes causally to hernia enlargement remains to be established.

**Figure 1:**
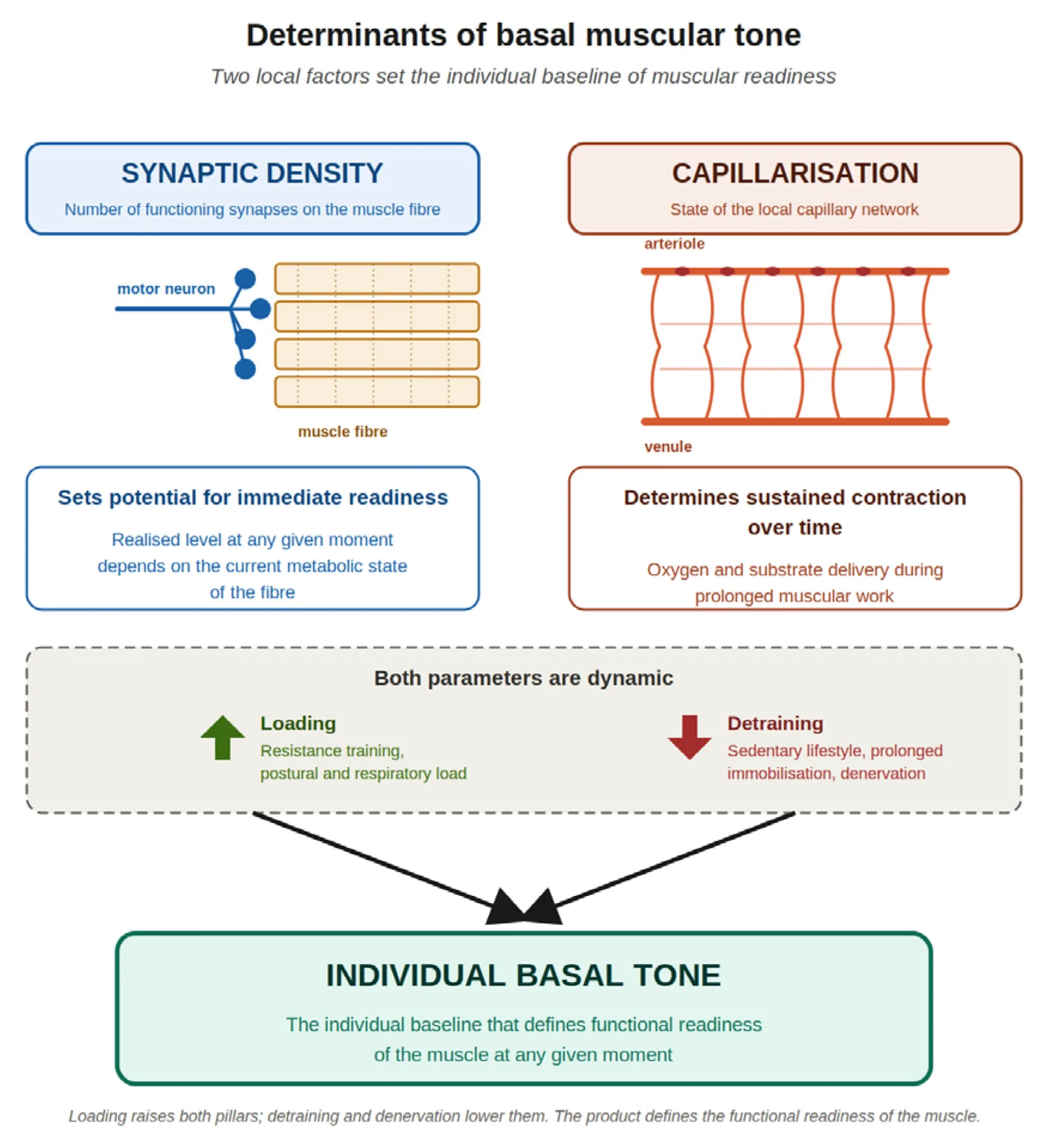
Determinants of basal muscular tone. Two local factors set the individual baseline: synaptic density, which determines the muscle’s potential for immediate readiness (with the realized level at any given moment modulated by the current metabolic state of the fiber), and capillarization, which determines how sustained that contraction can be over time. Both pillars are dynamic: they rise under loading and fall under detraining, immobilization or denervation, jointly defining the functional readiness of the muscle.

When the feedback loop is disrupted, the muscle fiber enters a state of denervation supersensitivity: acetylcholine receptor density increases, the depolarization threshold drops, and the contractile apparatus is remodeled toward slow myosin heavy chain isoforms [10,11,12,13]. Basal tone is determined by two local factors: the number of functioning synapses on the fiber (setting the potential for immediate readiness) and the state of the local capillary network (determining how sustained the contraction can be over time) (Figure 1).

### 3.2. Coordination of Synergistic Muscles: The Role of Intermuscular Synchrony

Functional protection of weak areas is provided not by the absolute strength of individual muscles, but by coordinated simultaneous contraction of all four muscular layers (external oblique, internal oblique, transversus abdominis, and rectus abdominis). The abdominal cavity functions as a biomechanical feedback system where the diaphragm and abdominal muscles act as antagonists, damping the peaks of dynamic IAP [6].

Intermuscular synchrony delivers the baseline stabilization. Muscles with highly asymmetric basal tone cannot provide a coordinated response to sudden elevation of IAP (cough, sneeze, straining) because their thresholds and activation rates differ. This difference in basal tone creates the prerequisite for discoordination: at the moment of peak load, some areas have already contracted while others have not, leaving a temporal gap where elevated IAP acts directly on a weak point that has lost its functional protection. Loss of intermuscular synchrony caused by differences in basal tone between synergistic muscles is a critical predisposing factor that is parallel and complementary to connective tissue weakness.

#### The Spine and the Abdominal Wall: A Famework of Discoordination

The thoracolumbar fascia, abdominal muscles, and IAP form a unified system that stabilizes the spine, providing up to 93% of the stabilizing effect on the lumbar segments [14]. Changes in IAP during postural tasks are mediated by coordinated activation of the diaphragm, pelvic floor muscles, and transversus abdominis [6].

When innervation is impaired, the abdominal cavity is transformed into a passive elastic reservoir whose walls can no longer damp pressure actively. Under reduced nerve conduction, contractile capacity and muscle mass are lost; in parallel, neurodystrophic processes involving aponeurotic tissues develop. Degenerative thoracolumbar spine disease, most common between the ages of 50 and 70, frequently overlaps with a sharp rise in hernia disease. Compressive neuropathy of the spinal roots in spondylotic radiculopathy decreases nerve stimulation of the abdominal muscles, reducing their excitability and basal tone. On the side where radiculopathy is clinically manifest, reduced neuromuscular activation may impair functional protection of the abdominal wall and could potentially contribute to conditions favoring hernia formation.

The resulting framework proposes a potential pathophysiological chain: connective tissue dysplasia → degenerative changes in the thoracolumbar spine → uneven compression of Th7–L1 roots → differences in impulse conduction velocity and loss of intermuscular synchrony → loss of the coordinated abdominal response to elevated IAP → increased susceptibility to hernia formation under provocative load. Each link of this chain is supported by separate work in neurology, biomechanics, and surgery. However, no direct study has been identified that links verified degenerative thoracolumbar spine disease with electrophysiologically confirmed discoordination of the abdominal muscles and subsequent hernia formation.

### 3.3. Muscle Memory and Pathological Adaptation During Prolonged Hernia Carriage

Basal tone is linked to muscle memory, which possesses a solid mechanistic basis: cellular (retention of myonuclei contributed by satellite cells during hypertrophy) and epigenetic (persistent changes in DNA methylation) [15,16,17,18]. A meta-analysis (147 studies) showed that with atrophy exceeding 30%, fibers lose myonuclei (standardized mean difference = −1.02; 95% CI −1.53 to −0.51; *p* < 0.001) [19]. DNA methylation proved more persistent: 7 weeks of training cause hypomethylation in promoters of growth-associated genes, and during detraining, the mass returns to baseline while the hypomethylation persists [20]. Negative muscle memory has shown that cells exposed in vitro to high doses of tumor necrosis factor alpha retain an epigenetic mark of the atrophic stimulus [21,22].

**Figure 2:**
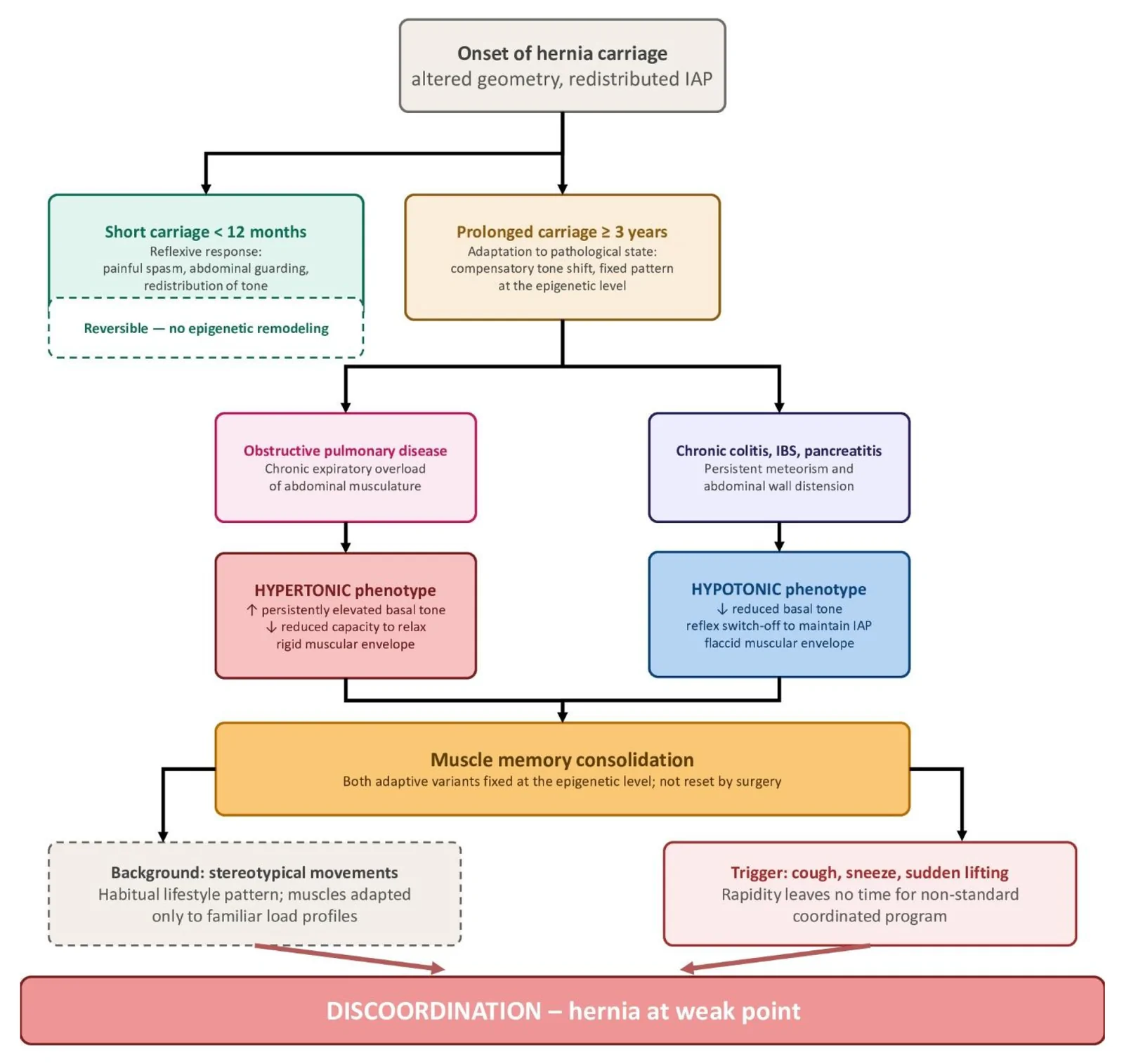
Proposed conceptual model of phenotypic divergence of the abdominal wall musculature with hernia carriage and comorbidity. Short-duration carriage (<12 months) is hypothesized to predominantly involve reflexive and potentially reversible responses, whereas prolonged carriage (≥3 years) is proposed to favor more persistent adaptive neuromuscular patterns. These time intervals represent working conceptual categories rather than prospectively validated biological thresholds. In the proposed model, obstructive pulmonary disease is associated with a predominantly hypertonic phenotype, whereas chronic gastrointestinal disorders with persistent meteorism are associated with a predominantly hypotonic phenotype. Muscle-memory mechanisms, potentially including epigenetic remodeling, are hypothesized to contribute to persistence of these adaptive patterns during prolonged carriage. Reduced adaptability to rapid unfamiliar loads (cough, sneeze, sudden lifting) may subsequently promote discoordinated contraction and increase susceptibility to herniation at mechanically vulnerable sites. The proposed relationships between hernia duration, muscle memory, epigenetic remodeling, and hernia formation remain hypothesis-generating and require prospective validation.

Within the proposed framework, we hypothesize that the duration of hernia carriage may influence the persistence and reversibility of adaptive neuromuscular patterns (Figure 2). Short-duration hernia carriage may predominantly be associated with reflexive and potentially reversible responses, such as protective spasm and guarding, whereas prolonged exposure to altered abdominal wall geometry, redistributed IAP, and compensatory changes in muscle tone may promote progressively more persistent neuromuscular adaptation. As a working conceptual model, we distinguish an early phase of hernia carriage (<12 months) from prolonged carriage (≥3 years), while recognizing that these time intervals have not been prospectively validated as biological thresholds. In the prolonged-carriage phenotype, mechanisms of muscle memory, including persistent epigenetic remodeling demonstrated in other skeletal-muscle contexts [15,16,17,18,19,20,21,22], may theoretically contribute to stabilization of the adapted contraction pattern. Whether such epigenetic consolidation occurs in the abdominal wall musculature of patients with ventral hernias, and whether it depends on the duration of hernia carriage, remain to be established directly.

In obstructive pulmonary disease, a phenotype with elevated basal tone and reduced capacity to relax develops; in gastrointestinal disorders with meteorism, the opposite hypotonic phenotype emerges. Within our conceptual model, these conditions illustrate two potential directions of neuromuscular adaptation during prolonged hernia carriage: a predominantly hypertonic and a predominantly hypotonic phenotype (Figure 2). We hypothesize that muscle memory may contribute to the persistence of either phenotype after prolonged adaptation, although this mechanism has not been directly demonstrated in patients with ventral hernias. Anatomical restoration alone may therefore not necessarily normalize a previously established neuromuscular pattern. Such persistent adaptation may reduce the capacity of the abdominal wall musculature to respond coordinately to rapid or unfamiliar loads, such as coughing, sneezing, or sudden lifting. Within the proposed framework, the resulting discoordination is hypothesized to increase susceptibility to herniation or postoperative functional failure (Figure 2).

### 3.4. Clinical Models of Abdominal Wall Denervation

A natural clinical model of segmental denervation is post-herpetic pseudohernia. An analysis of 34 cases showed that with involvement of the Th10–Th12 dermatome, a paresis of the corresponding segment develops, producing a hernia-like bulge without a fascial defect [23]. A review of 94 cases established that motor involvement in herpes zoster occurs in 0.5–5% of patients; the pseudohernia is usually self-limiting but requires differential diagnosis from true hernia [24]. In 12 of 13 patients, needle EMG or nerve conduction studies documented denervation potentials, and MRI showed hyperintensity of paraspinal muscles (involvement of the posterior branches) [25]. A prospective single-case assessment with ultrasound and surface EMG confirmed asymmetry of contraction and reduced activation on the affected side [26].

Beyond post-herpetic denervation, localized relaxation of the abdominal wall is described in other neuropathies: even with an intact fascial framework, impaired innervation can produce a clinically significant bulge. Four patients with acquired neurogenic wall weakness of diverse etiology (spinal meningioma, paralytic poliomyelitis, post-laminectomy denervation, diabetic thoracic radiculoneuropathy) have been described [27]; in three, proposed repair was deferred and the bulge resolved spontaneously. In 2025, two cases of pseudohernia with Th11–Th12 disc herniation and root compression were reported; CT excluded true hernia and treatment was conservative, with regression in 3–10 months [28]. Monoradiculopathy at Th11–Th12 from a non-calcified foraminal disc herniation produced pseudohernia in a 35-year-old man without comorbidity, with complete resolution on MRI at 6 months [29].

These observations share a common feature: localized loss of innervation of one or two segments—regardless of cause—can produce clinically significant abdominal wall dysfunction even in the absence of a fascial defect. These clinical models therefore provide indirect evidence that segmental denervation may compromise the functional competence of the abdominal wall. Within the proposed framework, we hypothesize that procedure-related denervation during component separation could similarly reduce the capacity of the affected muscle segments to participate in coordinated contraction, even when fascial continuity has been surgically restored. Whether such neuromuscular impairment contributes to limited functional recovery, altered abdominal wall sensation, chronic pain, or recurrence adjacent to a denervated segment has not been established and requires direct prospective investigation.

### 3.5. Botulinum Toxin and Component Separation as Examples of Iatrogenic Denervation

In 2026, six systematic reviews and meta-analyses (Table 1) devoted to preoperative BTA in ventral hernias have been published [30,31,32,33,34,35]. The 2021 meta-analysis demonstrated lateral muscle elongation of 3.2 cm per side (95% CI 2.0–4.3) and an increase in the fascial closure rate (RR 1.08; 95% CI 1.02–1.16) [30]. The 2025 meta-analysis with trial sequential analysis (TSA) showed that for the main clinical endpoints, the required information size has not been reached [35]. None of these reviews assessed the electromyographic (EMG) activity of the muscles after toxin washout; the time-course of basal tone recovery was never a study endpoint.

BTA blocks acetylcholine release and produces flaccid paresis; recovery proceeds through axonal sprouting over 12–16 weeks [36,37]. However, whether the time course of functional recovery differs according to the pre-injection basal tone of individual abdominal muscles has not been established. Within the proposed framework, we hypothesize that heterogeneous recovery of neuromuscular function could permit re-emergence of a pre-existing discoordinated contraction pattern after the toxin effect ceases; this possibility requires direct longitudinal electrophysiological evaluation.

Component separation techniques (ACS, PCS, TAR) address fascial mobilization but may also affect the neuromuscular integrity of the abdominal wall [38,39]. A 2025 cadaveric study clarified the topography of intercostal nerve entry into the rectus muscle [40], and a review confirmed the vulnerability of the 7th–12th intercostal nerve branches during subcostal approaches [41]; further anatomical characterization of posterior component separation with transversus abdominis release confirms relationships relevant to nerve preservation [42,43]. These anatomical data indicate that nerve branches may be vulnerable during component separation, although the extent and functional consequences of procedure-related denervation have not been established prospectively. Unlike BTA-associated chemodenervation, which is followed by axonal sprouting, division of a nerve branch may result in persistent denervation of its supplied territory. We hypothesize that such denervation could alter postoperative neuromuscular coordination and limit the capacity of the affected muscle to participate in coordinated retraining; however, the relationship between surgical denervation, muscle memory, and postoperative functional outcomes remains to be directly investigated.

Data from the neurology of skeletal muscle spasticity suggest that reduction in muscle tone after BTA does not necessarily translate into improved motor control and that directed rehabilitation may contribute to functional recovery [37]. Whether this principle is applicable to abdominal wall recovery after BTA in ventral hernia surgery has not been established. In this setting, BTA has demonstrated effects on muscle elongation and fascial closure [30,31,32,33,34,35], whereas restoration of coordinated neuromuscular function after toxin washout has not been directly investigated. Within the proposed framework, we therefore hypothesize that anatomical restoration and muscle elongation may not necessarily be accompanied by restoration of coordinated contraction. Whether phenotype-guided rehabilitation can improve neuromuscular recovery after BTA remains an open question and represents a target for prospective investigation.

### 3.6. Electrophysiological Evidence

A prospective surface electromyography (sEMG) study documented a significant reduction in the maximal force of contraction of the external and internal oblique muscles on the defect side, which was also bilateral (Table 2) [44,45]. A scoping review revealed extreme heterogeneity of assessment instruments and the absence of a standardized protocol [46], while studies of EMG limitations after BTA showed dissociation between functional computed tomography (CT) and single-point EMG [47].

Static CT records the musculature in apnea and does not reflect contractile reserve. Within the proposed framework, combining structural imaging with functional maneuvers (forced breathing, cough, simulated straining, and postural tasks) may provide a more informative characterization of the dynamic neuromuscular phenotype than static assessment alone. However, the optimal combination of functional maneuvers and electrophysiological or imaging modalities has not been established and requires prospective validation. Sarcopenia, which has been associated with worse outcomes after repair [48], may represent an additional factor relevant to neuromuscular readiness and could be evaluated in parallel in future studies.

## 4. Discussion

**Figure 3:**
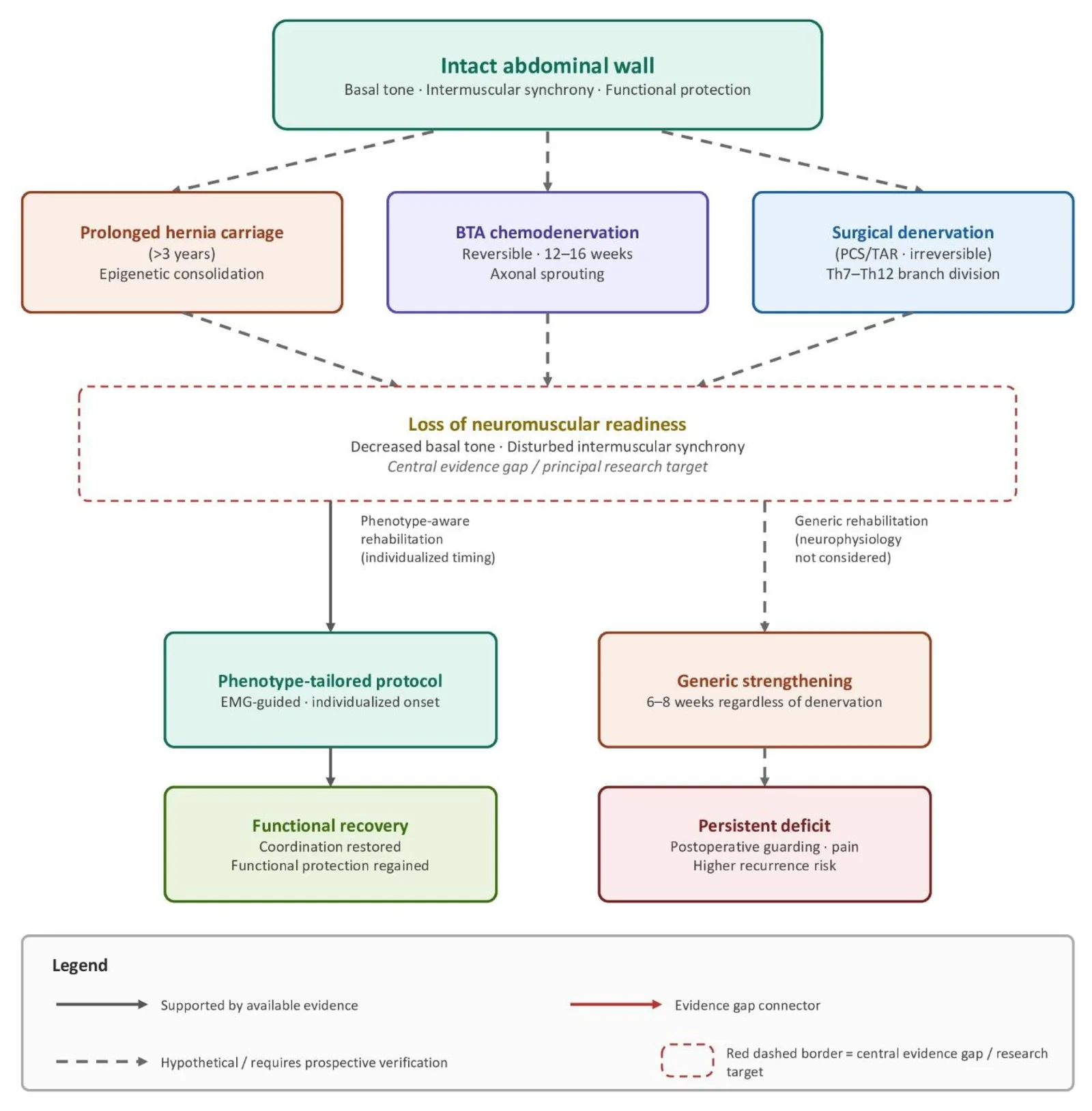
Conceptual model of neuromuscular readiness, denervation and rehabilitation pathways in abdominal wall hernia surgery. The intact abdominal wall (top) maintains basal tone, intermuscular synchrony and functional protection of weak areas. The model integrates three potential pathways that may alter neuromuscular readiness: prolonged hernia carriage, for which persistent adaptive contraction patterns potentially involving muscle-memory and epigenetic mechanisms are hypothesized; BTA-induced chemodenervation, followed by axonal sprouting over approximately 12–16 weeks; and potential procedure-related denervation during PCS/TAR. These pathways are proposed to converge on varying degrees of impaired neuromuscular readiness, although their relative contributions and interactions have not been established prospectively. The two rehabilitation pathways shown in the figure represent alternative conceptual scenarios rather than demonstrated clinical outcomes: phenotype-guided rehabilitation is hypothesized to facilitate functional neuromuscular recovery, whereas the possibility that rehabilitation not accounting for neuromuscular phenotype may contribute to persistent functional impairment, guarding, pain, or recurrence remains to be tested. Solid arrows indicate links supported by available evidence; dashed arrows indicate hypothetical links requiring prospective verification; the dashed red border identifies the central evidence gap and the principal research target. BTA, botulinum toxin type A; PCS, posterior component separation; TAR, transversus abdominis release.

The presented data allow the formulation of a position not articulated in the hernia literature up to now: the hernia patient comes to the surgeon not with an isolated fascial defect but with an integrated neuromuscular state of the abdominal wall that was shaped long before the operation (Figure 3). Basal tone, coordination of synergistic muscles and muscle memory together form an individual neuromuscular phenotype that is not recognized by existing preoperative protocols and is not taken into account in the choice of surgical strategy or in planning of rehabilitation. Within the proposed framework, we hypothesize that this phenotype may influence how the abdominal wall functions after anatomical integrity is restored.

Assessment of basal tone here differs fundamentally from a simple “good or poor” measurement. The patient’s tone corresponds to the phenotypic state at presentation, and the entire perioperative strategy must proceed from it. No two patients are alike: comorbidities, movement pattern, spinal innervation, and defect duration produce an individual neuromuscular profile. Moreover, the state changes within one patient—during the day (activity, emotion, food intake, breathing) and over longer periods depending on the preparatory regimen. The phenotype, therefore, cannot be captured by an assessment carried out months in advance: the functional status of the muscles must be characterized immediately before surgery, and tactics built from the real preoperative state rather than from an averaged characteristic. Existing algorithms ignore this, relying on defect size and volumetric relationships rather than on the functional state of the muscles that will operate after surgery.

Closure of the defect and placement of a mesh address the anatomical problem; muscle memory, consolidated over years of adaptation to the pathological state, is not reset in the operating room. At the first provocative stimulus, the muscles are again activated according to the prior pattern—which probably explains the disproportionate postoperative pain sometimes labeled an abdominal compartment syndrome: the anatomy has been restored, but the neuromuscular program has remained unchanged.

In this logic, BTA and component separation perform a surgical function—muscle elongation, reduction in tension and facilitation of fascial closure [30,31,32,33,34,35]—but their capacity to restore neuromuscular coordination has not been established. We hypothesize that, after the BTA effect ceases, recovery of muscle tone and neuromuscular function may permit re-emergence of a pre-existing discoordinated contraction pattern; however, this proposed sequence requires direct electrophysiological validation. The biomechanics of respiration aggravate the picture: under physiological conditions, the diaphragm and abdominal muscles damp IAP fluctuations in a coordinated rhythm, whereas after chemodenervation or separation, their reflex sequence is disrupted and the cavity behaves as a passive reservoir with raised baseline pressure at every cycle [6]. This does not alter the indications for these interventions but raises the hypothesis that phenotype-tailored neuromuscular retraining may have a role in postoperative functional recovery, a possibility that requires prospective evaluation.

The proposed framework yields practical consequences, each requiring prospective testing: (1) prehabilitation guided by the individual phenotype—assessment of basal tone, spinal status and hernia duration before surgery; (2) inclusion of multimodal neuromuscular evaluation (sEMG, functional CT or ultrasound-based contractility assessment) in the protocol, primarily in hernia carriage over three years, degenerative spinal disease and recurrence; (3) a preliminary conceptual model for rehabilitation differentiated by type of intervention (Table 3), presented as a hypothesis-generating framework rather than a validated clinical protocol: after BTA, delayed initiation by 12–16 weeks; after PCS/TAR, compensatory activation of preserved muscle groups; (4) postoperative rehabilitation as prehabilitation: closure of the defect does not mean the muscles have been rehabilitated—they must relearn coordinated contraction; (5) a prospective study with EMG mapping of the abdominal wall before and after BTA and PCS/TAR, correlating results with hernia duration, phenotype and functional outcomes. The results of such studies will inform the refinement of the proposed conceptual framework and, ultimately, the development of evidence-based clinical protocols.

The following framework (Table 3) is presented as a hypothesis-generating model derived from our conceptual synthesis. It is not intended as a clinical practice guideline. The proposed timelines and rehabilitation strategies require prospective validation through appropriately designed interventional studies. We acknowledge that the current evidence base does not support definitive recommendations regarding the optimal timing, intensity, sequence, or content of neuromuscular rehabilitation after ventral hernia repair; the framework is therefore offered as a structured approach for future investigation.

The proposed framework requires prospective verification: its clinical value will be determined by prospective studies showing whether preoperative neuromuscular phenotyping of the abdominal wall improves patient selection, defines the content of prehabilitation, and optimizes postoperative recovery.

### Limitations

The level of evidence supporting the proposed framework is heterogeneous (see Table 4). For propositions concerning basal tone and denervation, meta-analyses are available, but none contain neuromuscular endpoints. For discoordination in spinal pathology, only in silico models and indirect clinical analogies exist. For muscle memory in hernia carriage, fundamental reviews have been conducted in the context of sports physiology and age-related sarcopenia; no study has yet been performed in hernia patients. EMG-based evaluation is represented by two prospective studies and one scoping review without meta-analysis. The transfer of fundamental data to the clinical situation of hernia carriage is a conceptual extrapolation requiring direct clinical verification. The impossibility of assessing the transversus abdominis with sEMG limits the informativeness of the available electrophysiological data.

Several additional limitations of the present review should be acknowledged. The proposed neuromuscular framework is based on a conceptual synthesis of heterogeneous evidence rather than on direct prospective validation of the complete proposed pathophysiological sequence. Several components of the framework rely on extrapolation from adjacent fields or on evidence addressing individual links of the proposed mechanism rather than the mechanism as a whole. Accordingly, the temporal and causal relationships among altered basal tone, intermuscular discoordination, muscle memory, changes in abdominal wall biomechanics, and ventral hernia formation cannot be established from the available evidence. The rehabilitation strategies and timelines presented in Table 3 are likewise hypothesis-generating and should not be interpreted as evidence-based clinical recommendations. Their optimal composition, timing, intensity, and duration remain to be established in prospective interventional studies. In addition, title and abstract screening was performed by a single author, which may have increased the risk of selection bias, although full-text assessment and final inclusion decisions were made collegially by all authors.

**Table 4:** Evidence map of the proposed neuromuscular framework of abdominal wall hernia pathogenesis and recovery.

Block	Proposition	Key Sources	Evidence Status	What Is Supported	What Requires Prospective Testing
A	Chemodenervation and surgical denervation disturb the basal tone of the abdominal musculature	Meta-analyses [30,31,32,33,34,35]; BTA neurophysiology [36,37]; functional CT after BTA [47]	Supported by available evidence (surgical endpoints)/hypothesis only (neuromuscular endpoints)	Lateral muscle elongation; increased fascial closure rate; mechanism of axonal sprouting	Basal tone recovery after BTA (EMG dynamics); time to return of contractility; optimal rehabilitation window
B	Degenerative thoracolumbar spine disease may contribute to discoordinated contraction of the abdominal muscles and thereby increase susceptibility to hernia formation	FEA model [14]; biomechanics of IAP [6]; clinical observation	Indirectly supported	Separate links of the chain: spinal degeneration → root compression; impaired coordination → instability; load → defect	The whole chain: EMG mapping in patients with verified degenerative thoracolumbar spine disease and hernia
C	Prolonged hernia carriage may promote persistent adaptive patterns of muscular contraction, potentially involving mechanisms of muscle memory and epigenetic remodeling	Reviews of muscle memory [15,16,17,18,21,22]; meta-analysis [19]; epigenetics [20]	Indirectly supported	Epigenetic DNA methylation persists after detraining; negative muscle memory in vitro	Any study of muscle memory in the context of hernia carriage; correlation of hernia duration with postoperative guarding
D	A standardized neuromuscular assessment of the abdominal wall is lacking	sEMG studies [44,45]; scoping review [46]; CT + EMG [47]	Partially supported	Bilateral reduction in strength in hernia patients; heterogeneity of methods; insufficiency of single-point EMG	Development and validation of a multimodal neuromuscular assessment protocol for surgical purposes

Operational definitions of these categories are provided in the Methods section; they represent an evidence-mapping tool developed for this conceptual review and not a formal evidence-grading system. FEA, finite element analysis; IAP, intra-abdominal pressure; sEMG, surface electromyography; CT, computed tomography; EMG, electromyography; BTA, botulinum toxin type A. Evidence status: supported by available evidence/partially supported/indirectly supported/hypothesis only.

## 5. Conclusions

Modern ventral hernia surgery has achieved progress in mesh technology, standardization of fascial closure, and refinement of component separation, yet the neuromuscular layer of the abdominal wall has remained outside clinical planning. The proposed framework of neuromuscular readiness (Figure 3) suggests that patients with ventral hernias may present with an individual neuromuscular phenotype shaped by basal tone, coordination of synergists, and muscle memory. Placement of a mesh and closure of the fascia solve the anatomical problem; the neuromuscular problem remains unsolved if rehabilitation does not take this phenotype into account. We hypothesize that successful treatment may extend beyond fascial closure and may benefit from restoration of coordinated neuromuscular function through individualized rehabilitation. This hypothesis requires prospective validation in appropriately designed interventional studies before it can be translated into clinical practice. Prospective validation—EMG mapping, differentiated protocols of pre- and postoperative rehabilitation, and correlation with the phenotype—will determine to what extent this approach improves long-term functional outcomes.

The conclusions of the present review and the proposed neuromuscular framework are primarily based on evidence concerning ventral abdominal wall hernias and should be interpreted within this context. Groin hernias have distinct anatomical and pathophysiological mechanisms and were not the focus of the present review. Nevertheless, we hypothesize that selected principles of neuromuscular phenotyping, individualized prehabilitation, and phenotype-guided rehabilitation may also be relevant to other types of abdominal wall hernias. This possibility should be regarded as a separate hypothesis and requires dedicated investigation before any broader applicability of the framework can be established.

## Figures and Tables

**Table 1:** Evidence from systematic reviews and meta-analyses of preoperative botulinum toxin A in ventral hernia repair: surgical endpoints versus neuromuscular endpoints.

Author, Year	Studies (*n*)	Patients (*n*)	Lateral Muscle Elongation, cm/Side (95% CI)	Fascial Closure	Recurrence	Wound Complications	EMG Assessment	Basal Tone Recovery Assessed	TSA Performed	Overlap with Previous Reviews
Timmer et al. (2021)	23	995	3.2 (2.0–4.3; I^2^ = 0%)	RR 1.08 (1.02–1.16; I^2^ = 0%)	0.03 (0.01–0.06)	Descriptive	No	No	No	Reference base
Dias et al. (2023)	7	261	4.11 (3.76–4.46; I^2^ = 27%)	Descriptive	0.03 (0.01–0.06)	SSI 0.06; SSO 0.12	No	No	No	High overlap
van Rooijen et al. (2021)	20	541	Not pooled separately	0.94 (0.89–0.98)	0.03 (0.01–0.06)	Descriptive	No	No	No	Partial overlap (BTA + PPP)
Mejía-Saavedra et al. (2025)	5	869	Not assessed	RR 0.95 (0.90–1.01; *p* = 0.10)	RR 1.02 (0.64–1.49)	RR 0.66 (0.50–0.88)	No	No	No	Updated evidence base
Barretto et al. (2024)	12	676	Descriptive	Descriptive	Descriptive	Descriptive	No	No	No	Partial overlap
Florencio de Mesquita et al. (2025)	4 (PSM)	Mixed	Not assessed	Pooled (PSM)	Pooled (PSM)	Pooled (PSM)	No	No	Yes (RIS not reached)	PSM design—added analytic value

RR, relative risk; CI, confidence interval; SSI, surgical site infection; SSO, surgical site occurrence; PPP, progressive preoperative pneumoperitoneum; PSM, propensity-score matched; TSA, trial sequential analysis; RIS, required information size.

**Table 2:** Electromyographic and functional studies of the abdominal wall in hernia patients.

Author, Year	Design	*n*	Assessment Method	Muscles Studied	Main Finding	Methodological Validity in Hernia Patients	Type of Relevance to Neuromuscular Framework
Sreenath et al. (2016)	Prospective controlled	87	sEMG	EO, IO bilaterally	Reduced force on the hernia side and contralateral side (*p* < 0.001)	Not established in hernia population	Direct clinical evidence
Joppin et al. (2023)	Prospective	≈40	sEMG	EO, IO	Reduced abdominal muscle power in hernia patients	Not established	Direct clinical evidence
Tomazini Martins et al. (2019)	Prospective post-BTA	56 (11 with CT+EMG)	Functional CT + single-point EMG	EO, IO, TrA	Heterogeneous paralysis; dissociation between CT and EMG findings in 11 cases	Single-point EMG shown to be insufficient	Methodological evidence
Choi et al. (2021)	Case report	1	sEMG + ultrasound + needle EMG	EO, IO, TrA, RA	Denervation potentials and asymmetric activation in herpes zoster pseudohernia	Confirmed against ultrasound	Denervation model
Shen et al. (2026)	Scoping review	18 studies	Dynamometry, sEMG, clinical tests	Various	No standardized protocol identified	Heterogeneity of instruments documented	Evidence mapping/scoping evidence

sEMG, surface electromyography; EO, external oblique; IO, internal oblique; TrA, transversus abdominis; RA, rectus abdominis; CT, computed tomography; BTA, botulinum toxin type A.

**Table 3:** Conceptual matrix for testing perioperative rehabilitation hypotheses across denervation scenarios.

Parameter	Post-BTA Without Component Separation	Post-PCS/TAR Without BTA	Post-BTA + PCS/TAR (Combined)	Mesh Repair Without Denervation
Type of denervation	Pharmacological (chemodenervation)	Surgical (division of Th7–Th12 branches)	Combined	None
Reversibility	Reversible (axonal sprouting)	Irreversible in the divided territory	Partially reversible	Not applicable
Expected time to reinnervation	12–16 weeks after injection	None in the divided territory	12–16 weeks (BTA). None in the divided territory.	Not applicable
Timing for prospective comparison	Delayed-start arm at 12–16 weeks	Timing stratified by functional assessment	Stepwise arm starting at ≥16 weeks	Current-practice comparator starting at 6–8 weeks
Rehabilitation focus for comparison	Coordination retraining versus strength loading	Compensatory recruitment of preserved muscle groups	Staged combination of both approaches	Conventional abdominal strengthening comparator
Preoperative EMG in study design	Phenotype stratification after prolonged hernia carriage (>3 years)	Baseline mapping of denervation	Baseline mapping and phenotype stratification	Selective baseline assessment
Hypothesized effect on motor pattern	Temporary disruption with retraining after recovery	Persistent disruption in surgically denervated territory	Combined disruption with greater retraining demand	Pattern preserved with possible postoperative guarding

The entries define candidate study variables and should not guide current clinical care. BTA, botulinum toxin type A; PCS, posterior component separation; TAR, transversus abdominis release; EMG, electromyography. All proposed pathways are conceptual and require prospective clinical validation; they should not be interpreted as clinical recommendations.

## Data Availability

No new data were created or analyzed in this study. Data sharing is not applicable.
